# 1.L. Workshop: Building capacity for a resilient and healthy post-COVID health workforce

**DOI:** 10.1093/eurpub/ckac129.039

**Published:** 2022-10-25

**Authors:** Organised by: EUPHA-HWR, WHO Europe, Chair persons: Ellen Kuhlmann (EUPHA-HWR), Tomas Zapata (Denmark)

## Abstract

**Background:**

The COVID-19 pandemic added new challenges to health workforce resilience. High workload, increased risk of COVID infection and continuing stress threaten the health and wellbeing of healthcare workers (HCWs) and increase existing (gendered) inequalities, thus reinforcing health labour market problems. Investment in the health workforce and prioritisation of the health and wellbeing needs of healthcare workers in health policy and pandemic recovery plans is therefore called for by the WHO, the European Union and others. However, health systems and policymakers across countries have not sufficiently understood the importance of HCWs.

**Objectives:**

This workshop brings health workforce needs onto the health policy agenda. It seeks to build capacity for a resilient and healthy health workforce, setting the focus on protection and preparedness of HCWs and their individual wellbeing, and reduction of gendered inequalities. The following major questions will be addressed: What do we know about COVID-19 and the health and wellbeing of HCWs? What skills are needed to respond to new demands? What policy approaches are available to improve protection, preparedness and mental health support of HCWs? How to integrate gender equality and the situation of migrant/refugee HCWs in these approaches, and finally build capacity for effective health workforce governance and its implementation.

The workshop introduces novel results drawn from European comparative research and country case studies. It connects policy and practice, as well as health system and individual HCW needs. A number of important policy recommendations are emerging from the research. (1) The health and wellbeing of the health workforce must be addressed systematically across all levels of health policy and governance, including the organisation settings. (2) Health systems must take responsibility and leadership in responding to health workforce needs. (3) Important lessons to be learned from HCWs’ experiences should guide future health workforce policy and pandemic recovery plans. (4) Participatory governance and inclusion of HCWs must be strengthened, including gender equality and diversity.

The workshop will stimulate critical debate and improve knowledge exchange across countries and between researchers. It will contribute to strengthen resilience of the health workforce and health systems, and to build back better after COVID-19 in a fair and equitable manner.

**Key messages:**

• Health workforce needs and mental health support must become policy priorities and addressed as part of health system resilience.

• Action has to be taken to strengthen participatory health workforce governance sensitive to new needs.

